# Ethnopharmacology of five flowers herbal tea, a popular traditional beverage in Hong Kong and South China

**DOI:** 10.1186/s13002-024-00674-z

**Published:** 2024-03-15

**Authors:** Kwun-Tin Chan, Hoi-Yan Wu, Wing-Yee Tin, Paul Pui-Hay But, Sidney Chin-Hung Cheung, Pang-Chui Shaw

**Affiliations:** 1 Li Dak Sum Yip Yio Chin R & D Centre for Chinese Medicine, The Chinese University of Hong Kong, Shatin, Hong Kong China; 2 Institute of Future Cities, The Chinese University of Hong Kong, Shatin, Hong Kong China; 3 School of Life Sciences, The Chinese University of Hong Kong, Shatin, Hong Kong China; 4 Department of Anthropology, The Chinese University of Hong Kong, Shatin, Hong Kong China

**Keywords:** Five Flower Tea, Herbal tea, DNA barcoding, Ethnopharmacology, Formulation of traditional herbal medicine

## Abstract

**Background:**

It has been a long-standing tradition of using herbal tea for preventive and therapeutic healthcare in Hong Kong and South China and Five Flowers Tea is one of the most popular herbal teas. Based on the principle of traditional Chinese medicine, the pharmacological functions are to clear heat and dispel dampness in the body. Heat and dampness are thought to contribute to a range of health problems, especially during the hot and humid season in South China and Hong Kong. The most prevalent herbs in the formula contain bioactive compounds including flavonoids, alkaloids and terpenoids, which have a wide range of pharmacological properties including anti-inflammation, antivirus, antidiarrhoea, antibacteria, and antioxidation. However, with the composition varies widely, the ethnopharmacological benefits described may not be delivered uniformly. This study is to provide a comprehensive analysis on the composition of the Five Flowers Tea sold in Hong Kong and investigate the rationale behind the selection of herbs used in the formula. This study also provides information on the variation and quality of the Five Flowers Tea in the market.

**Methods:**

Thirty-three Five Flowers Tea samples were collected from various locations in Hong Kong. The size, texture, colour and organoleptic properties were documented. Macroscopic and molecular authentication methods were employed to identify the individual components.

**Results:**

Macroscopic identification revealed there were 23 herbs belonging to 18 plant families. The most prevalent herb was *Bombax ceiba* L., followed by *Chrysanthemum morifolium*. Ten adulterants and the existence of insect *Lasioderma serricorne* were confirmed by DNA barcoding techniques.

**Conclusion:**

This study employed a comprehensive approach to authenticate the herbs in Five Flowers Tea samples collected from various locations in Hong Kong. Macroscopic and molecular methods were used to identify the herbs and adulterants. The findings revealed the varied composition in Five Flowers Tea and the occurrence of adulterants in some samples. This shows that quality assurance of Five Flowers Tea is essential for the effective use of this popular folk medicine.

**Supplementary Information:**

The online version contains supplementary material available at 10.1186/s13002-024-00674-z.

## Introduction

Herbal tea, also called “cooling tea”, “herbal beverage” or “liáng chá”, is widely used for preventive and/or therapeutic healthcare in Hong Kong and South China. Herbal tea is a decoction with a range of herbal ingredients prepared according to the ethnomedical experiences of indigenous and local people [1]. The use of herbal tea is also regarded as a popular primary healthcare system in many countries [2].

The history of drinking herbal tea in China can be traced back to the Tang dynasty (AD618-907), proposed in the ancient Chinese literature *Beiji Qian Jin Yao Fang* [3]. Southern China, especially in Lingnan region, has a rich tradition of drinking herbal tea [4]. The tradition of drinking herbal tea in Hong Kong has a long history, yet the documented evidence pertaining to its origins and development remains scarce. In 1869, a Hong Kong government gazette ordinance mandated that ships transporting Chinese labours overseas must carry Chinese medicinal herbs in proportion to the number of passengers [5]. The requirement of preparing herbal tea for passengers during their voyage, signifies the popular utilization of herbal tea for ailment treatment and illness prevention in the nineteenth-century Hong Kong. In 1949, the government announced the requirement for a license to sell herbal tea [6], officially regulating the herbal tea business. Until now, herbal tea shops have been a fixture of Hong Kong landscape and still very much a part of daily life. Herbal tea has been inscribed in the national list of Intangible Cultural Heritage in 2006 [5].

Hong Kong has a subtropical climate with a wet–dry season [7]. Both written in Qing Dynasty (1644–1911), *Lingnan fengwu ji* (a record of the customs of Guangdong) *and Chronicle of Sun-On County* described the unfavourable climate and environmental conditions and frequent epidemic plagues happened in Hong Kong. From the perspective of Chinese Medicine, hot and wet weather are thought to contribute to a series of health problems. Exogenous heat and dampness evils attack the body, resulting in the abnormal consumption of qi (basic element that constitutes the cosmos) in the body and impairment of body fluids. In particular, the interstices and mysterious mansion on the skin remain open in summer and spring time to release the internal heat, and hence people are susceptible to the illness. To prevent the above conditions and alleviate the symptoms, people gradually learnt to use local botanical resources for making herbal tea. They boiled herbs for decoctions to treat a range of health problems from infectious diseases, cardiovascular disease to digestive disorders [1, 8]. The practice of drinking herbal tea builds up the immune system and helps to adopt the harsh environmental conditions.

So far, over 400 plant species have been used in producing herbal teas in southern China [9, 10]. The formulae of herbal teas vary in different regions [4]. The most iconic and representative herbal tea is Five Flowers Tea. This decoction is mostly consumed during the spring and summer months. Five Flowers Tea can be found globally because it has developed a widespread consumer base. The growing Chinese populations in cities like Toronto, London, Los Angeles and Sydney have boosted demand for traditional herbal remedies. These markets provide ingredients for expat communities and locals wanting to explore health traditions. The primary functions are to clear heat and expel dampness which helps to alleviate symptoms including stomach ache, abdominal bloating, indigestion and diarrhoea [1, 11]. There is no official formula of Five Flowers Tea in the Chinese Pharmacopeia. On the other hand, some of the local publications suggested that the mainstream formula of Five Flowers Tea includes the flower of *Lonicera japonica* (honeysuckle), *Plumeria rubra* (frangipani), *Pueraria lobata* (kudzu), *B.* *ceiba* (tree cotton) and *Sophora japonica* (Japanese pagoda tree) in equal weight ratio. Apart from the stated herbs, herbal tea shops and distributors may add some other herbs to reinforce the heat clearing and dampness expulsion function [11, 12]. The lack of standardisation in Five Flowers Tea formula poses problems. Different producers may use substitutes or adulterated ingredients due to the availability issues or to cut cost. This inconsistency in formulation can undermine the therapeutic claims and safety. So far, information and analysis on the composition of Five Flowers Tea and the rationale of using the herbs are lacking. We therefore set forth to analyse the authenticity, diversity and quality of Five Flowers Tea found in the Hong Kong market.

Given this, the objectives of this study are as follows: (1) to comprehensively analyse and document the botanical compositions of Five Flower Tea samples from Hong Kong using macroscopic and molecular identification methods, (2) to investigate the common and varying herbal ingredients across samples, and identify any adulterants present, (3) to evaluate compositional differences and variations between samples of different retail sources, (4) to determine whether the variation in compositions could impact the traditional uses and purported health benefits relied upon by the public and (5) to provide standardisation recommendations and contribute novel data to support the use and quality control of herbal tea.

## Methods

### Study area and sample collection

Hong Kong lies at the southern part of China, bordered by the South China Sea on all sides except the north. The territory consists mostly of Hong Kong Island, Kowloon Peninsula and New Territories. The study area has rugged relief and marked variations, with very limited flatland, comprising mostly woodland, shrubland and grassland [13]. Hong Kong has a subtropical climate in which the wet–dry seasonal change is apparent; greater variation in temperature and humidity can be observed when compared to other subtropical areas [7].

**Fig. 1:**
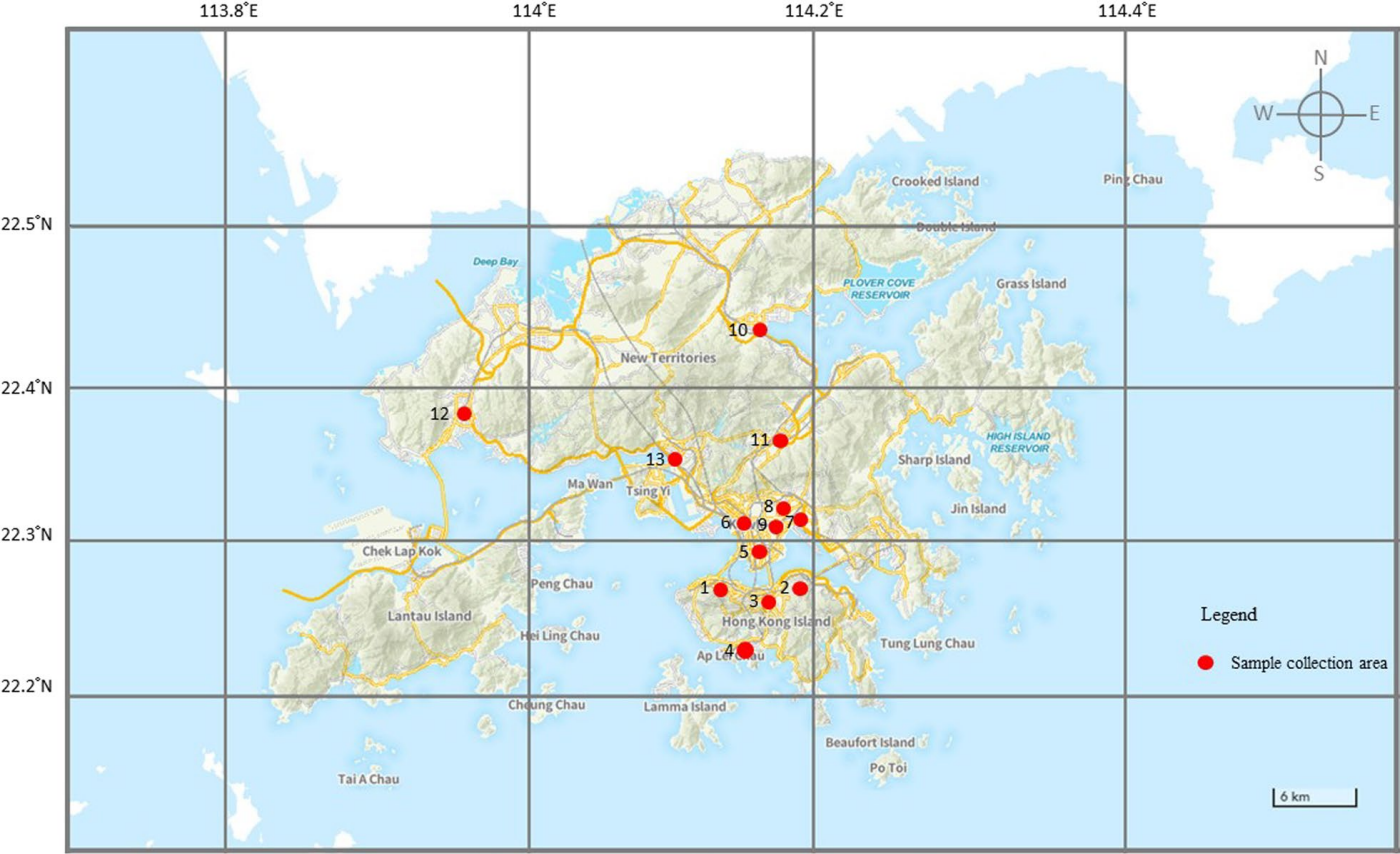
Map of the study area for the collection of sample in Hong Kong. Investigated locations: 1. Central and Western District; 2. East District; 3. Wan Chai District; 4. South District; 5. Yau Tsim Mong District; 6. Sham Shui Po District; 7. Kwun Tong District; 8. Wong Tai Sin District; 9. Kowloon City District; 10. Tai Po District; 11. Sha Tin District; 12. Tuen Mun District; 13. Tsuen Wan District (photo courtesy: GeoInfo Map, The Government of Hong Kong SAR)

Sample collection was conducted between 2021 and 2023 in Hong Kong. Thirty-three bags of Five Flowers Tea were collected from local grocery stores, herbs retailers, market stalls and Chinese medicine pharmacies from 13 districts. The sampling locations were organised by administrative region, and each sample was assigned a unique voucher number. Table 1 and Fig. 1 detail the district of purchase and corresponding voucher numbers for the Five Flowers Tea samples collected. Claims and instructions given by the suppliers, total weight and size of the parcel and species of herbs were recorded (Additional file 1).

**Table 1:** Sample collection locations and voucher numbers

No	Location	Sample voucher
1	Central and Western District	HK01 HK11
2	East District	HK02 HK03
3	Wan Chai District	HK04 HK05 HK06 HK07 HK08 HK09 HK12
4	South District	HK10
5	Yau Tsim Mong District	KL02
6	Sham Shui Po District	KL01 KL03 KL04 KL05
7	Kwun Tong District	KL06 KL07 KL08
8	Wong Tai Sin District	KL09
9	Kowloon City District	KL10
10	Tai Po District	NT01 NT02 NT05
11	Sha Tin District	NT03
12	Tuen Mun District	NT04 NT06 NT07 NT08
13	Tsuen Wan District	NT09 NT10 NT11

### Voucher specimen collection and macroscopic examination

The voucher specimen of each botanical sample was collected and deposited in Li Dak Sum Yip Yio Chin R&D Centre for Chinese Medicine at the Chinese University of Hong Kong (Additional file 1). Macroscopic identification of the Five Flowers Tea samples includes shape, size, surface, texture, cross section and type of fracture. Organoleptic examination including colour, odour and taste was carried out by comparing with the herbs listed in the Chinese Pharmacopoeia [14].

### Molecular authentication

Total DNA was extracted from 2 g of each dried sample using a broad-spectrum plant rapid genomic DNA extraction kit (Biomed, China) according to the instructions of the kit manufacturer. DNA was visualized by electrophoresis in a 1.5% agarose gel, and images were collected (Gel Doc EZ system, Bio-Rad, Hercules, CA). DNA concentration was measured by Nanodrop spectrophotometer (DeNovix, Wilmington, DE).

Universal primers (COI-F: 5’-GGT CAA CAA ATC ATA AAG ATA TTG G-3’ and COI-R: 5’-TAA ACT TCA GGG TGA CCA AAA AAT CA-3’) were used for the amplification of the COI (Cytochrome Oxidase I) region. Another pair of universal primers (psbAF: 5’-GTT ATG CAT GAA CGT AAT GCT C-3’ and trnHR: 5’- CGC GCA TGG TGG ATT CAC AAT CC-3’) were used for amplification of the psbA-trnH region. A pair of universal primers (ITS2F: 5’- ATG CGA TAC TTG GTG TGA AT-3’ and ITS3R: 5’-GAC GCT TCT CCA GAC TAC AAT-3’) were used for amplification of the ITS2 region. The 30μl PCR reaction mixes included 21.4μl of ultrapure water, 3μl of DNA template, 1.2U of DNA polymerase, 3μl of 10X PCR buffer, 0.6μl of deoxynucleoside triphosphates (10nM), 0.3μl of each primer (10μM) and 1.2μl of 50mM MgSO_4_. Amplifications were carried out using a thermal cycler (Veritipro 96W). The thermal regime consisted of an initial denaturation at 94°C for 5 min; 30 cycles of 94 °C for 1 min, 56 °C for 1 min and 72 °C for 1.5 min; and a final extension at 72 °C for 7 min. PCR products were visualised on 1.5% agarose gel. Amplicons of the correct sizes were selected and purified for subsequent sequencing by BGI, China. The obtained sequences were aligned by ClustalW multiple alignment in MEGA11. The identities of species were identified via BLASTN, NCBI in GenBank database at their highest similarity.

### Data analysis

Data analysis was carried out to evaluate the composition of different ingredients of Five Flowers Tea samples using Microsoft Excel. Voucher number, name of herbal materials, voucher code, weight, total weight of the package, percentage of herbal materials, indication and administration, condition and remarks are listed in Additional file 1. Excel was also used for statistical analyses. Sequences generated from samples in this study were assembled and aligned using CodonCode aligner for comparison and checking.

## Results

### Five Flowers Tea samples

**Fig. 2:**
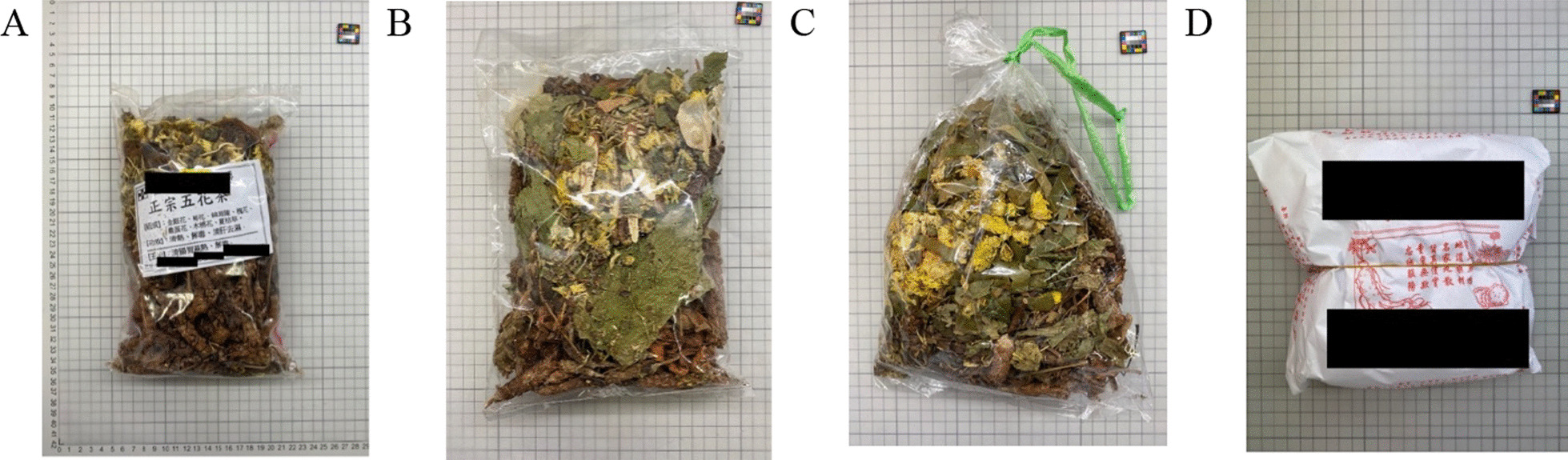
Packaging styles of Five Flowers Tea samples. **A** HK07: Transparent plastic bag with a piece of note stating their brand, name of the tea, ingredients and functions. **B** KL03: Transparent plastic bag closing with heat seal. **C** KL07: Transparent plastic bag tying by nylon string. **D** HK08: Wrapped by paper printed with company name and address

We collected 33 Five Flowers Tea samples from different locations in Hong Kong. Most were packed by transparent plastic bags, with some of them having logos of the shops. A sample was wrapped by paper in the traditional way. Heat seal was the major way to close the plastic bag. Other ways included tying by string and a knot directly on the bag (Fig. 2).

Among the 33 samples, 14 were labelled with description including the names of the herbal tea, herbs included or functions. Four were labelled with the brand name and 14 did not have label. One sample was labelled as Heat Dampness Clearing Tea instead of Five Flowers Tea.

### Profiles of Five Flowers Tea samples

**Fig. 3:**
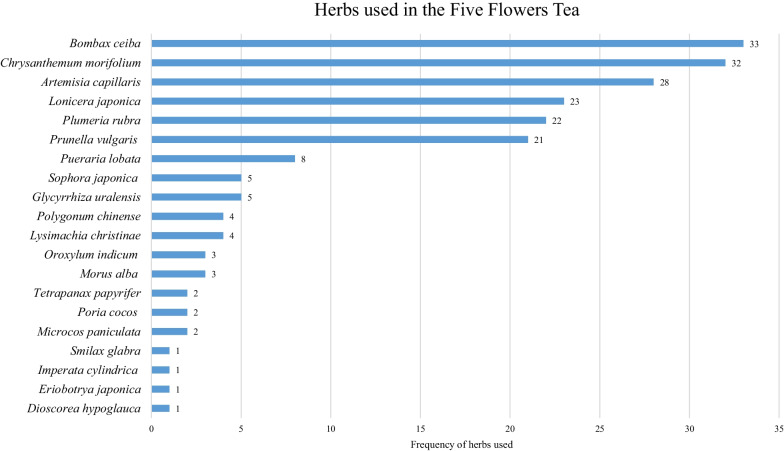
Herbs used in the Five Flowers Tea. Number denotes the frequency of herbs used

A total of 23 herbs in 18 families were identified by macroscopic identification (Additional file 2). Components of the herbs in each sample are listed in Additional file 1. Figure 3 shows that the most prevalent herb is *B. ceiba*, followed by *C. morifolium*. The third place is *Artemisia capillaris*, a herb collected in non-flowering period. It is popular for the shop owners to change some ingredients in the Five Flowers Tea formula to lower the cost or increase the potency.

### Composition of parcel samples

Additional file 1 and Table 2 illustrates the composition of herbs in the different parcel samples. HK07, HK12, KL04, KL05 and NT04 contain *B. ceiba*, *P. rubra*, *C. morifolium*, *L. japonica* and *S. japonica*, which are herbs in the classical formula. However, the weights of *L. japonica* and *S. japonica *in HK07 were exceptionally low compared to other samples. Two extra herbs, *A. capillaris* and *P. vulgari*s, were found. For HK12, the weight of *B. ceiba* was around 45 g, triple to that other four ingredients *P. rubra*, *C. morifolium*, *L. japonica* and *S. japonica*, which were around 15 g. For KL04, the weight of *B.* *ceiba* was over 40 g. For sample KL05, *B.* *ceiba* outweighed all the herbs by 2 to 10 times, followed by an extra herb *A. capillaris*. For NT04, the quantity of *B.* *ceiba* and *P. rubra* was higher than the rest of the herbs.

**Table 2:** Composition of Five Flowers Tea samples

	MMH	JDH	JH	JYH	HH	GH	YC	XKC	HTM	SY	QCZ	GC	TC	TFL	FL	JYH	BX	BMG	BZY	PPY
HK01	60	5	4	2			15	12												
HK02	62	3	2				13													
HK03	35	5	6	1			15													
HK04	47	9	15				12	23				7								
HK05	28		14	6			22	27			6			24						
HK06	45		6	1			19	19	74											
HK07	15	13	14	3	2		21	25												
HK08	51	23	16	5			29													
HK09	51	20	19	15			19													
HK10	107		20	7			42	9												
HK11	64	19	15	14		14	21													
HK12	46	15	14	14	16															
KL01	84		2	1		5	45	2												
KL02	35	12	13	5			5	19		6		7			12					
KL03	36	2	7	3			14	16	16	9	4	4								
KL04	41	13	27	2	7															
KL05	43	16	7	4	3	5	20													
KL06	62						14				5					6		8	10	
KL07	17		15				20	20	9			5								
KL08	27	15	20				43	26												21
KL09	28		13					24								14	11		34	
KL10	50	12	11			9	32													
NT01	31	5	7			4	25	15				10								
NT02	54	3	10	2			36	20												
NT03	34	10	9	6		6	32	28												
NT04	34	36	24	25	24															
NT05	37		6	2			19	10	16							25				
NT06	13		2				8	17		79			1		10					
NT07	35		11				55	32					1							
NT08	38	6	9	6		5	23	13												
NT09	42	3	14	4			45	26												
NT10	19		11	4		9		5												
NT11	56	18	19	7			36									17				

Number in the table denotes weight in gram

MMH: Bombax ceiba, JDH: Plumeria rubra, JH: Chrysanthemum morifolium, JYH: Lonicera japonica, HH: Sophora japonica, GH: Pueraria lobata, YC: Artemisia capillaris, XKC: Prunella vulgaris, HTM: Polygonum chinense, SY: Morus alba, QCZ: Oroxylum indicum, GC: Glycyrrhiza uralensis, TC: Tetrapanax papyrifer, TFL: Smilax glabra, FL: Poria cocos, JYH: Lysimachia christinae, BX: Dioscorea hypoglauca, BMG: Imperata cylindrical, BZY: Microcos paniculate, PPY: Eriobotrya japonica

Non-traditional herbs prevailed in both HK06 and NT06. For HK06, 41.3% of the weight of the whole package was dried whole plant of *Polygonum chinense* L. For NT06, 55.9% was *Morus alba* L.

### DNA barcoding for the authentication of herbs

We used DNA barcoding technique to identify the suspected adulterants. Barcoding region psbA-trnH was used followed by BLASTN search from GenBank database for the authentication of *L. japonica* and *A. capillaris*. The result suggested that four samples had *Lonicera macranthoides* instead of *L. japonica*. For the suspected adulterant of *A. capillaris*, BLASTN research indicated that one sample was *Potentilla chinensis*, while another was *Potentilla discolor*. ITS2 region was sequenced to examine four doubtful samples of *Lysimachia christinae*, and it was found that all were *Desmodium styracifolium*. Table 3 illustrates the ten found adulterants.

**Table 3:** Summary of identification of suspected adulterants using BLASTN search from GenBank

Voucher number	Species claimed	DNA barcoding region	Species identification using BLASTN	Max percent identity
HK09	*Lonicera japonica*	psbA-trnH	*Lonicera macranthoides*	100
HK10	*Artemisia capillaris*	ITS2	*Potentilla chinensis*	99.01
HK11	*Lonicera japonica*	psbA-trnH	*Lonicera macranthoides*	97.61
KL05	*Lonicera japonica*	psbA-trnH	*Lonicera macranthoides*	100
KL06	*Lysimachia christinae*	ITS2	*Desmodium styracifolium*	100
KL09	*Lysimachia christinae*	ITS2	*Desmodium styracifolium*	100
NT05	*Lysimachia christinae*	ITS2	*Desmodium styracifolium*	100
NT07	*Artemisia capillaris*	ITS2	*Potentilla discolor*	99.71
NT11	*Lysimachia christinae*	ITS2	*Desmodium styracifolium*	100
NT05	*Lonicera japonica*	ITS2	*Lonicera macranthoides*	97.79

### Procedures for decoction generation

A total of 16 parcels were advised to bring up a boil, and all the parcels were advised to brew with different volumes of water, time length and level of heat. It was suggested that other ingredients can be added to increase the potency or flavour. Rock sugar and *Siraitia grosvenorii* (Lo Han Kuo) were the most prevalent optional additives to Five Flowers Tea, recommended by five store owners. For KL02, slab sugar but not rock sugar was suggested. Store owner of KL07 suggested to add *L. japonica.* Additional file 1 summarizes the recommended procedures for preparing the decoctions.

### Storage conditions and identity of insects

**Fig. 4:**
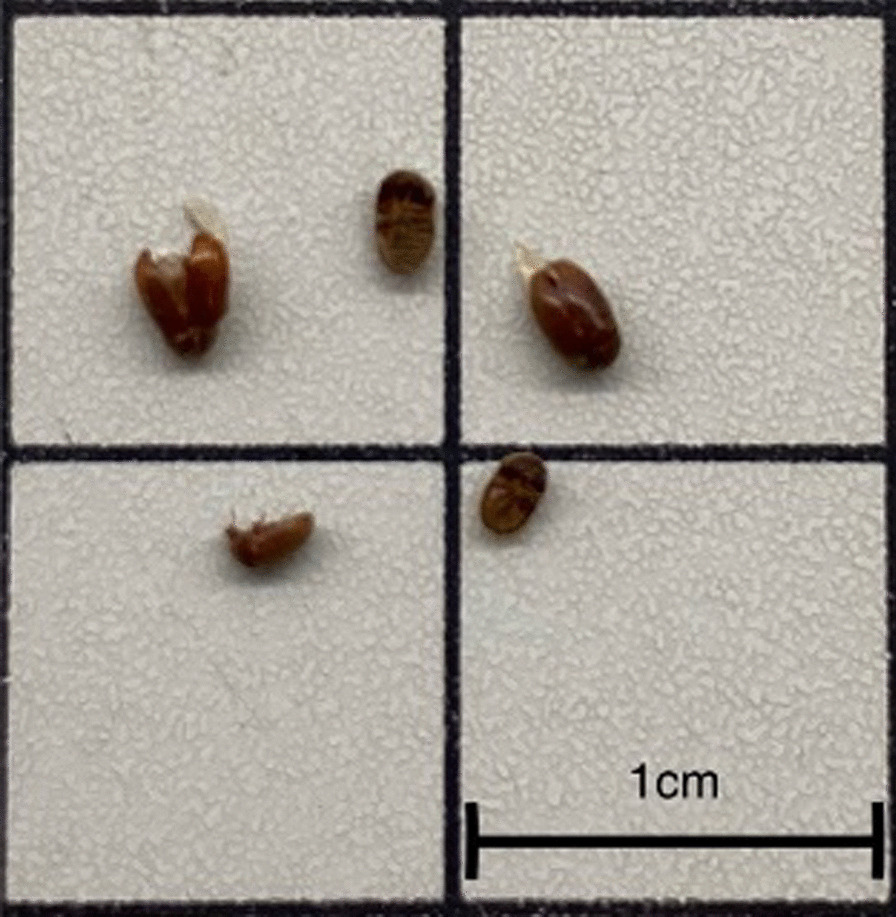
Image of insects collected from sample HK08 as an example

All 12 samples that contained insects were discovered to be moist. We found that all collected insect samples were *L. serricorne * (Fig. 4), as revealed by sequencing the COI region and compared with the data in BOLD Identification System (BOLD-IDS) or BLAST search in GenBank.

## Discussion

### Variation of five flowers tea formula

Etymologically, the term Wu Hua Cha can be directly translated into Tea with Five Flowers. The most used formula is based on the combination of five herbs, *B. ceiba, P. rubra, C. morifolium, L. japonica* and *S. japonica*. These five herbs possessed the function of heat clearing, dampness draining and detoxifying. The herbs would act on intestinal area and liver. In terms of pharmacological effect, most of the five herbs possess the function of anti-inflammatory, antibacterial, antiviral, antidiarrhoeal and diuretic effect, aligning with the ethnomedical claims.

In this study, we only found eight samples comprised of the five floral herbs. Some herbal shop owners mentioned that some herbs are cut to reduce the cost. Herbal tea is a grassroot remedy in respond to the expensive fee for consulting a clinician. Therefore, there is often a trade-off between the cost and potency of the herbal tea. *Prunella vulgari*, for example, is a cheaper alternative to other expensive herbs like *Pueraria lobata* and *S. japonica*. Over half of the parcels (63.6%) contain it. *Prunella vulgari* is renowned in the heat clearing function and as a major ingredient in another popular herbal tea Xiasangju [15].

*A. capillaris* was found in 28 out of 33 parcels (84.8%) (Fig. 3). This herb is not a component in the classic formula. Also, the herb is not in the flowering stage. The harvesting season of *A. capillaris* is between March and April [16]. It is believed that during this period, the medicinal properties of *A. capillaris* are at their strongest for clearing heat and draining dampness, surpassing its effectiveness when collected during the flowering period.

*A. capillaris* was used to substitute *S. japonica.* According to the principle of Chinese medicine, *A. capillaris* has a greater dampness draining ability, but *S. japonica* is better in heat clearing. Some studies suggested that *A. capillaris* may have hepatoprotective, anticancer and anti-inflammatory properties [17–19]. Therefore, herbalists may preferentially select *A. capillaris* for its superior dampness dispelling capacity.

### DNA authentication of herbs

Herbal remedies often face issues of adulteration due to the difficulty of visually identifying authentic herbs from fraudulent substitutes. The visual appearance of closely related herbal species can be highly similar, making organoleptic identification difficult for those without specialized knowledge of the subtle morphological differences. This confusion has led to many instances of adulteration, such as *D. styracifolium* being used as *L. christinae*, *Potenilla* species substituting for *A. capillaris* and *Lonicera confuse* being used in place of *L. japonica*.

NA barcoding method was performed to identify the herbs used in Five Flowers Tea parcel. DNA barcoding is a molecular technique used to identify species based on species-specific differences in a short, standardised fragment of nucleus and organelle DNA from a specific gene or genome region. That DNA fragment is then compared to a reference DNA database to determine the identity [20].

This method can precisely and rapidly identify species from various raw materials. In plant identification, the common barcode regions used are ITS2 and psbA-trnH. The region ITS2 (Internal Transcribed Spacer 2) is located in the nuclear genome, between 5.8S and 28S rRNA genes [21]. The region psbA-trnH is in the non-coding region of the chloroplast genome. Due to their high variability, the region can serve as a distinctive marker for a particular species [22].

The COI region, which is located in the mitochondrial genome of eukaryotic organisms, was used to identify insects due to its significant level of intra-specifically similarity and inter-specifically variation [23].

Using DNA barcoding technique, we detected the substitution of *L. japonica*, *A. capillaris* and *L. christinae* by* L. macranthoides*, *Potentilla* species and *D. styracifolium*, respectively. This may pose risks of having a reduced therapeutic efficacy or unintended side effects.

### The pros and cons of using the substitutes and adulterants

The first herb in question was *L. japonica.* While *L. japonica* and *L. macranthoides* show similar pharmacological efficacy as evidenced by their medicinal usage history, the Chinese Pharmacopeia (2005 Edition) lists them separately. Both *L. japonica* and *L. macranthoides* exhibit low toxicity [24]. There have been many clinical trials conducted on *L. japonica*. In contrast, clinical investigations on *L. macranthoides* are limited. Both *L. japonica* and *L. macranthoides* share similar properties, including being categorized as cold in nature, sweet in flavour and having comparable therapeutic functions and applications. *L. macranthoides* is commonly cultivated and used as a substitute for *L. japonica* in China. Despite having similar chemical profiles and pharmacological effects, *L. macranthoides* offers a higher yield and lower production cost. This makes *L. macranthoides* an economical alternative to *L. japonica* [25].

The second herb in question was *A. capillaris*. Studies have shown that *A. capillaris* has greatly benefited the treatment of many diseases such as liver inflammation, cirrhosis and liver cancer, contributing to the wider development and advancement of traditional Chinese medicine [26]. *A. capillaris* and its active compounds have antioxidant, anti-inflammatory, antifibrotic, antiviral and anticancer effects. This also supports its traditional uses for treating liver and viral disorders as well as cancer [27–29].

We found that *Potentilla* species was used to replace *A. capillaris* in Five Flowers Tea. *Potentilla* species have been noted for their medicinal functions that are akin to those of *A. capillaris*, although there are variations between the herbs in terms of chemical profiles and effectiveness in treating certain conditions. *Potentilla* species have been used in traditional medicine, particularly in Chinese medicine, for a long time to treat ailments such as diarrhoea, hepatitis, rheuma, scabies and for detoxification purposes [30]. *A. capillaris* was found to be adulterated by *Potentilla* species, in particular *P. discolor* and *P. chinensis*. The aerial and underground parts of *P. discolor* have been used to treat inflammation, wound, cancer, infection caused by bacteria, fungi, and viruses, diarrhoea and diabetes mellitus [31]. There have been no reports of toxic effects during the long history of using *Potentilla* species and their extracts in traditional medicine [30].

Substituting *A. capillaris* by *P. discolor* and *P. chinensis* may be appropriate due to the pharmacological properties of the two latter herbs. *A. capillaris* is indicated for dispelling dampness due to its bitter and pungent flavour. *P. discolor* and *P. chinensis*, with their stronger cooling properties, are more suitable for resolving excessive heat conditions compared to *A. capillaris*.

The third herb in question was* L. christinae*. *L. christinae* is a traditional herb that possesses heat-clearing, diuretic, detumescent and detoxifying properties [32]. The adulterant *D. styracifolium* is widely used in traditional Chinese medicine for its diuretic and heat-clearing properties and for the management of urinary calculi, cardiovascular and cerebrovascular disorder and hepatitis [33].

From the Chinese medicine perspective, *L. christinae* and *D. styracifolium* have some similarities but also distinct differences in their indications and usage. Both herbs are valued for their diuretic and heat-clearing properties, which help resolve accumulated dampness heat and dissipate pathogenic factors. However, *L. christinae* focuses on dispelling dampness heat in liver and gallbladder. It is indicated for conditions that involve pathogenic factors affecting the hepatobiliary system. In contrast, *D. styracifolium* focuses more on dispelling dampness heat in the urinary bladder and kidney for urological conditions.

*L. christinae* and *D. styracifolium* therefore should be used interchangeably as they are for treating different types of conditions.

Herbal tea poisoning incidents have occasionally been reported, typically resulting from the use of toxic adulterants [34, 35]. In 2003, a local individual sought help after experiencing symptoms of poisoning. *Datura metel*, a plant known to contain toxic tropane alkaloids, was discovered in the brewing residue of the Five Flowers Tea [36]. The presence of adulterants in herbal tea can pose significant dangers to public health, ensuring the safety and quality of herbal tea products is therefore crucial.

### Instructions on brewing and precautions

Instructions for brewing the tea differed among the different shops. The time, quantity of water, procedure of brewing and heat level vary (Additional file 1).

Furthermore, indications on the contradictions are simple and not mentioned in every parcel. For instance, sample HK10 stated that it is suitable for everyone regardless of their health condition. Sample NT02 suggested that people who are of “cold” constitution can only drink half of a bowl. G6PD deficient patients were suggested to avoid taking *L. japonica* [37, 38]. Of the 33 samples surveyed, only one parcel provided notice that people with G6PD deficiency should avoid using the product.

Apart from the potential danger of G6PD, a retailer has mentioned that pregnant women should avoid taking the Five Flowers Tea. However, a survey has indicated that more than half of Chinese pregnant women living in Hong Kong have taken Chinese herbal medicine, including Five Flowers Tea and *L. japonica*, for improving their gastrointestinal and digestive health. The use of Chinese herbal medicine during pregnancy did not appear to affect the condition, incidence or severity of jaundice in both normal and G6PD-deficient infants at birth [39]. The herbs used in Five Flowers Tea are not included in the List of Restricted Chinese Herbal Medicines for Pregnant Women [40].

### *Lasioderma serricorne* infestation and quality control

*L. serricorne* was first reported and identified on tobacco in France in 1848 [41]. It is currently globally distributed and exceptionally prevalent in tropical and subtropical areas and is the most destructive pest affecting a wide variety of materials derived from both plants and animals [42]. It has long been recognised as the dominant pest species that infests stored Chinese medicinal materials, resulting in significant economic loss [43]. In our study, 12 out of 33 Five Flowers Tea samples were contaminated by *L. serricorne.*

The larvae of *L. serricorne* can cause significant damage by feeding on herbal tea leaves, resulting in reduced quality and value. Some conditions give rise to the growth of *L. serricorne.* Firstly, since herbal tea parcels are stored in compacted form, they provide an ample food source for the beetle to feed on. Secondly, the beetles like to hide away from bright light and low humidity. The best temperature range and relative humidity for rapid development of *L. serricorne* are 29–35 °C at 75% [44]. All our samples found with insects were moist. Hong Kong is warm and humid, not to mention the hot and humid storage conditions in warehouses and wet markets.

Thirdly, packaging material and styles would be another issue. The adult stage of the beetle is capable of biting through the packaging material [45]. In our study, different packaging styles are found (Fig. 2). Insect infestation is higher when the herbs are wrapped in a piece of paper or put in a transparent plastic bag with opening tied with a nylon. *L. serricorne* can easily penetrate the paper wrap, and paper wrapping cannot protect the herbs from the humidity. Nylon string tied on the bag opening is easily loosened, creating an entry point for the insect and moisture to enter.

The presence of contaminated and gnawed herbs, as well as the excrement, cast-off skin and other products, can pose a menace to the health of consumers. *L. serricorne*, in particular, has been linked to canthariasis, a human-enteric infection [46]. Cases of intestinal canthariasis have been reported in China and Malaysia [46, 47].

Effective pest management methods include phosphine fumigation, CO_2_ treatment and radiation treatments and high temperature. In recent years, pest management has put more emphasis on friendly to the environment and cost-effectiveness [48]. For instance, the package can be treated with natural repellents that prevent *L. serricorne* from consuming the contents [49]. Examples of such repellents include crude leaf extracts of Tecoma stans and Datura metel, as well as various plant essential oils such as shiso oil and savory oil [50]. For storage, multilayer packaging is an alternative to applying pesticide or temperature control warehouse. *L. serricorne* was found to get through polyethylene, polypropylene and polyester [51]. Thicker packaging material and multilayer packaging better deterred the spreading of the insect [45].

### The interplay of cultural and environmental factors in the composition and evolution of Five Flowers Tea

In our study, we observed a diverse range of ingredients and proportions, with no unique recipe found. Several factors contribute to the variation in herbal tea recipes, including medicinal effects, Chinese philosophy, culture and environments.

Chinese philosophy emphasizes the interaction between human being and outside world and so is the traditional Chinese medicine. Factors such as individual person, time and place should be taken into account. In the holistic approach, each factor influences the others, and any adjustments should be considered in the overall context. We previously mentioned the impact of local climate and environment on the choice of herbs. For instance, locals brew “cold medicine” to get rid of heat-related ailments in Hong Kong’s hot and wet weather. Locals would consume specific herbal teas or Chinese medicine during different solar terms or seasons. For example, illness prevention teas are favoured in spring, Five Flowers Tea is for the hot summer, while soothing herbal teas are popular in autumn [5]. The proportions of ingredients in Five Flower Tea can also be adjusted for year–round consumption, not limited to the summer season.

Individual constitution and nature are factors leading to vast variations in herbal tea recipes. The proportions and the selection of herbs differ based on individual needs. When buying samples, vendors suggested different brewing methods and additives to the herbal tea in order to suit individuals need as well (Additional file 1).

The ingredients of Five Flowers Tea in Hong Kong are influenced and limited by the resources, local culture and habit. Recent studies indicated that not all the five flowers are readily available, leading to the brewing of herbal tea with fewer ingredients, and once the number of the ingredients down to *B. ceiba* alone [52]. The flexibility in Five Flowers Tea composition reflects its evolution from a specific herbal tea recipe to a cultural symbol, expanding the definition of Five Flowers Tea to a kind of drink which provides similar medicinal effects using various herbs with flowers.

The emerging needs of society, such as the occurrence of pandemics like COVID-19, also shaped the development of herbal tea. While standardisation of Five Flowers Tea can instil the confidence of consumers, certain variations may be beneficial for the medicinal efficacy.

In this study, the Five Flowers Tea samples were from vendors in Hong Kong. As herbal tea is also popular in South China, it will be interesting to expand the area of sample collection, to compare the quality and use of this medicinal product in nearby regions. The identification of herbs in this study is by organoleptic and DNA sequences. These sequences are either from our previous work [53] or GenBank. The matching of these two independent approaches can reveal the herb identity to high accuracy. We are constructing a DNA database of herbal tea material from authenticated samples, which will further enhance the confidence of authentication in the future.

## Conclusion

Five Flowers Tea has been consumed for prevention and therapeutic purposes in Southern China for centuries. This work provides a systematic analysis on the composition and quality of Five Flowers Tea parcels sold in Hong Kong. We have found the use of variety of herbs, adulterants, irregular instructions and insect infestation, poses significant risk to public health. A better quality control of this popular herbal tea is needed. Also, with a standardised formulation with recommended variations can consumers be confident that this culturally significant herbal remedy delivers its attributed effects.

### Supplementary Information

**Additional file 1:** Information on the purchased Five Flowers Tea Samples.

**Additional file 2:** Herbal tea ingredients identified in Five Flowers Tea samples and their pharmacological properties.

## Data Availability

The datasets used and/or analysed during the current study are available from the corresponding author on reasonable request.

## Abbreviations

28S: 28S ribosomal RNA gene
5.8S: 5.8S ribosomal RNA gene
*A. capillaris*: Artemisia capillaris
BLAST: Basic Local Alignment Search Tool
BOLD: Barcode of Life Data System
*B. ceiba*: Bombax ceiba
CO_2_: Carbon dioxide
COI: Mitochondrial cytochrome oxidase subunit 1 gene
*C. morifolium*: Chrysanthemum morifolium
*D. styracifolium*: Desmodium styracifolium
DNA: Deoxyribose nucleic acid
ITS2: Internal transcribed spacer 2
*L. japonica*: Lonicera japonica
*L. christinae*: Lysimachia christinae
*L. **macranthoides*: Lonicera macranthoides
*L. serricorne*: Lasioderma serricorne
*P. chinensis*: Potentilla chinensis
*P. discolor*: Potentilla discolor
*P. rubra*: Plumeria rubra
*P. vulgaris*: Prunella vulgaris
PCR: Polymerase chain reactions
psbA-trnH: Intergenic spacer between photosystem II protein D1 gene and tRNA-His gene
