# Supplementary material for: Ethnopharmacology of five flowers herbal tea, a popular traditional beverage in Hong Kong and South China
Source: J Ethnobiol Ethnomed. 2024 Mar 15;20:36. doi: 10.1186/s13002-024-00674-z (PMC10943788; doi:10.1186/s13002-024-00674-z)
Supplement: Supplementary file 1 — Additional file 1: Information on the purchased Five Flowers Tea Samples. [file 13002_2024_674_MOESM1_ESM.pdf]

Additional file 1: Information on the purchased Five Flowers Tea Samples

Voucher Number	Herbal material in the parcel [14, 54]	Botanical voucher code	Weight of the herbal material (g)	Total weight of the package (g)	Percentage of each herbal material (%)	Indication and administration	Condition of the parcel and remark
HK01	*Bombax ceiba* L.	T5774	60.17	141.64	42.48	1. Brew over medium heat with 20 bowls of water for 45 minutes<br><br>2. Can add sugar optionally to enhance the soothing effect	
	Plumeria rubra L.	T5775	4.7		3.32		
	Chrysanthemum morifolium Ramat.	T5776	4.22		2.98		
	Lonicera japonica Thunb.	T5777	2		1.41		
	Artemisia capillaris Thunb.	T5778	15.09		10.65		
	Prunella vulgaris L.	T5779	12.38		8.74		
HK02	*Bombax ceiba* L.	T5780	62.26	83	75.01	Bring to a boil over high heat and brew over low heat for 45 minutes	Reduce the cost by providing four flowers only
	Plumeria rubra L.	T5781	3		3.61		
	Chrysanthemum morifolium Ramat.	T5782	2.33		2.81		
	Artemisia capillaris Thunb.	T5783	12.91		15.55		
HK03	*Bombax ceiba* L.	T5784	35.11	75.93	46.24	Bring to a boil with 5 bowls of water over high heat and brew over low heat for 45 minutes	Fragmentary and moist herbs, insects found, marks of biting
	Plumeria rubra L.	T5785	5.05		6.65		
	Chrysanthemum morifolium Ramat.	T5786	5.88		7.74		
	Lonicera japonica Thunb.	T5787	1.44		1.9		
	Sophora japonica L.	T5788	14.6		19.23		

HK04	*Bombax ceiba* L.	T5789	46.81	131.18	35.68	1. Boil up with 8 bowls of water for 20 minutes<br><br>2. Can add *Siraitia grosvenorii* (lo han kuo) to enhance the soothing effect	Moist herbs, insects found
	Plumeria rubra L.	T5790	9.48		7.23		
	Chrysanthemum morifolium Ramat.	T5791	15.4		11.74		
	Artemisia capillaris Thunb.	T5792	12.32		9.39		
	Prunella vulgaris L.	T5793	23.47		17.89		
	Glycyrrhiza uralensis Fisch.	T5794	6.56		5		
HK05	*Bombax ceiba* L.	T5795	28.23	142.31	19.84	Boil with 8 bowls of water for 45 minutes until 3-4 bowls left	Label as tea of clear heat and drain dampness
	Chrysanthemum morifolium Ramat.	T5796	14.46		10.16		
	Lonicera japonica Thunb.	T5797	5.69		4		
	Artemisia capillaris Thunb.	T5798	21.66		15.22		
	Prunella vulgaris L.	T5799	27.44		19.28		
	Oroxylum indicum (L.) Vent.	T5800	6		4.22		
	Smilax glabra Roxb.	T5801	24.44		17.17		
HK06	*Bombax ceiba* L.	T5802	45.45	178.73	25.43	1. Brew over medium heat with 10 bowls of water for 60 minutes<br><br>2. Add ginger or dried tangerine peel to adjust the cold nature of the tea	
	Chrysanthemum morifolium Ramat.	T5803	5.95		3.33		
	Lonicera japonica Thunb.	T5804	0.68		0.38		
	Artemisia capillaris Thunb.	T5805	19.47		10.89		
	Prunella vulgaris L.	T5806	18.56		10.38		
	Polygonum chinense L.	T5807	73.83		41.31		
HK07	*Bombax ceiba* L.	T5808	15.15	109.11	13.89		

	Plumeria rubra L.	T5809	12.63		11.58	1. Bring to a boil with 5 and half bowls of water over high heat and brew over low heat for 45-60 minutes until 2 and a half bowls left<br><br>2. Ginger should be added if the drinker is of cold constitution	
	Chrysanthemum morifolium Ramat.	T5810	14.25		13.06		
	Lonicera japonica Thunb.	T5811	2.88		2.64		
	Sophora japonica L.	T5812	2.48		2.27		
	Artemisia capillaris Thunb.	T5813	20.85		19.11		
	Prunella vulgaris L.	T5814	25.07		22.98		
HK08	*Bombax ceiba* L.	T5815	50.68	145.62	34.8	1. Brew over medium heat with 8-9 bowls of water for 30 minutes until 3 bowls of water<br><br>2. Not suitable for people with cold constitution	Moist herbs, insects found
	Plumeria rubra L.	T5816	22.92		15.74		
	Chrysanthemum morifolium Ramat.	T5817	15.98		10.97		
	Lonicera japonica Thunb.	T5818	4.75		3.26		
	Artemisia capillaris Thunb.	T5819	29.06		19.96		
HK09	*Bombax ceiba* L.	T5820	50.75	139.78	36.31	Boil for around 30 minutes depends on the number of drinkers	Moist herbs, insects found
	Plumeria rubra L.	T5821	20.27		14.5		
	Chrysanthemum morifolium Ramat.	T5822	18.72		13.39		
	Lonicera confusa DC.	T5823	14.92		10.67		
	Artemisia capillaris Thunb.	T5824	19.19		13.73		

HK10	*Bombax ceiba* L.	T5825	106.7	204.55	52.16	1. Boil for around 30 minutes with 10-12 bowls of water<br><br>2. Rinse the herbs after open<br><br>3. Can boil together with wax gourd as soup<br><br>4. Add rock sugar after boiling to enhance the soothing effect<br><br>5. Suitable for everyone regardless of their body constitution	
	Chrysanthemum morifolium Ramat.	T5826	20.3		9.92		
	Lonicera japonica Thunb.	T5827	7.07		3.46		
	Potentilla chinensis Ser.	T5828	41.8		20.44		
	Prunella vulgaris L.	T5829	8.76		4.28		
HK11	*Bombax ceiba* L.	T5830	64.4	172.97	37.23	1. Brew over medium heat with 10-16 bowls of water until 7 bowls of water for 45 minutes	
	Plumeria rubra L.	T5831	18.71		10.82		
	Chrysanthemum morifolium Ramat.	T5832	14.6		8.44		
	Lonicera confusa DC.	T5833	14.32		8.28		
	Pueraria lobata (Willd.) Ohwi	T5834	13.71		7.93		
	Artemisia capillaris Thunb.	T5835	20.81		12.03		

						2. Add *S. grosvenorii* for lung soothing effect	
HK12	*Bombax ceiba* L.	T5836	46.15	121.17	38.09	Boil for 30 minutes with 6-7 bowls of water	The shop owner emphasis the authenticity of his Five Flowers Tea recipe
	Plumeria rubra L.	T5837	15.37		12.68		
	Chrysanthemum morifolium Ramat.	T5838	13.95		11.51		
	Lonicera japonica Thunb.	T5839	14.45		11.93		
	Sophora japonica L.	T5840	15.64		12.91		
KL01	*Bombax ceiba* L.	T5841	83.97	162	51.83	1. Brew for at least 45 minutes.<br><br>2. Optional to add rock sugar and brown slab sugar	Moist herbs, insects found
	Chrysanthemum morifolium Ramat.	T5842	2.05		1.27		
	Lonicera japonica Thunb.	T5843	0.68		0.42		
	Pueraria lobata (Willd.) Ohwi	T5844	4.56		2.81		
	Artemisia capillaris Thunb.	T5845	45.35		27.99		
	Prunella vulgaris L.	T5846	1.68		1.04		
KL02	*Bombax ceiba* L.	T5847	34.84	119.44	29.17	1. brew over medium heat with 8 bowls of water in 30-45 minutes<br><br>2. Optional to add brown slab sugar but not rock sugar	1. Consider *P. vulgaris and A. capillaris* as floral herbs<br><br>2. Moist herbs, insects found<br><br>3. *P. cocos* is a fungus
	Plumeria rubra L	T5848	12.22		10.23		
	Chrysanthemum morifolium Ramat.	T5849	13.43		11.24		
	Lonicera japonica Thunb.	T5850	5.33		4.46		
	Artemisia capillaris Thunb.	T5851	5.19		4.35		
	Prunella vulgaris L.	T5852	18.8		15.74		
	Morus alba L.	T5853	6.13		5.13		
	Glycyrrhiza uralensis Fisch.	T5854	7.25		6.07		
	Poria cocos (Schw.) Wolf	T5855	11.6		9.71		

KL03	*Bombax ceiba* L.	T5856	35.88	141.05	25.44	1. Rinse the herbs after open<br><br>2. Brew over medium heat with optimum amount of water<br><br>3. Optional to add brown slab sugar to extinguish the liver fire	The stall owner claimed that it is a variation of Five Flowers Tea, in which mixing Xiasangju tea together to generate a greater effect.
	Plumeria rubra L.	T5857	1.51		1.07		
	Chrysanthemum morifolium Ramat.	T5858	6.89		4.88		
	Lonicera japonica Thunb.	T5859	2.68		1.9		
	Artemisia capillaris Thunb.	T5860	14.34		10.17		
	Prunella vulgaris L.	T5861	16.1		11.41		
	Polygonum chinense L.	T5862	16		11.34		
	Morus alba L.	T5863	8.67		6.15		
	Oroxylum indicum (L.) Vent.	T5864	4.44		3.15		
	Glycyrrhiza uralensis Fisch.	T5865	4.18		2.96		
KL04	*Bombax ceiba* L.	T5866	41.44	100.94	41.05	1. Brew over medium heat with 10 bowls of water for 30 minutes<br><br>2. Optional to Add *S. grosvenorii*	The stall owner emphasized the dampness draining ability of *S. japonica* and the suitability of this tea for everyone
	Plumeria rubra L.	T5867	13.33		13.21		
	Chrysanthemum morifolium Ramat.	T5868	26.67		26.42		
	Lonicera japonica Thunb.	T5869	2.37		2.35		
	Sophora japonica L.	T5870	6.62		6.56		
KL05	*Bombax ceiba* L.	T5871	43.05	113.2	38.03	Brew the herbs with 6 bowls of water until 3 bowls left	
	Plumeria rubra L.	T5872	15.77		13.93		
	Chrysanthemum morifolium Ramat.	T5873	6.8		6.01		

	Lonicera confusa DC.	T5874	4.35		3.84		
	Sophora japonica L.	T5875	2.58		2.28		
	Pueraria lobata (Willd.) Ohwi	T5876	4.72		4.17		
	Artemisia capillaris Thunb.	T5877	20.46		18.07		
KL06	*Bombax ceiba* L.	T5878	61.94	151.29	40.94	1. Boil for 60 minutes<br><br>2. Amount of water can be adjusted according to the number of drinkers	Moist herbs especially *B. ceiba* L., lots of insects found
	Artemisia capillaris Thunb.	T5879	13.55		8.96		
	Oroxylum indicum (L.) Vent.	T5880	4.96		3.28		
	Desmodium styracifolium (Osh.) Merr.	T5881	5.62		3.71		
	Imperata cylindrical (Linn.) P. Beauv.	T5882	8.25		5.45		
	Microcos paniculata L.	T5883	10.3		6.81		
KL07	*Bombax ceiba* L.	T5884	16.94	151.05	11.21	1. Bring up a boil with 9 bowls of water and brew over low heat for 60 minutes<br><br>2. Add *L. japonica* and *S. grosvenorii*	Suitable for everyone regardless of their body constitution
	Chrysanthemum morifolium Ramat.	T5885	14.82		9.81		
	Artemisia capillaris Thunb.	T5886	19.57		12.96		
	Prunella vulgaris L.	T5887	20.03		13.26		
	Polygonum chinense L.	T5888	8.68		5.75		
	Glycyrrhiza uralensis Fisch.	T5889	4.59		3.04		
	Eriobotrya japonica (Thumb.) Lindl.	T5890	21.22		14.05		
KL08	*Bombax ceiba* L.	T5891	26.66	163.85	16.27	Boil with 6 bowls of water for 30-40 minutes until 3 bowls of water left	Addition of *A. capillaris* is to enhance the dampness draining performance of the tea
	Plumeria rubra L.	T5892	14.77		9.01		
	Chrysanthemum morifolium Ramat.	T5893	20.25		12.36		
	Artemisia capillaris Thunb.	T5894	43.01		26.25		

	Prunella vulgaris L.	T5895	25.58		15.61		
KL09	*Bombax ceiba* L.	T5896	27.58	149.1	18.5	1. Brew with 8-10 bowls of water for 45 minutes<br><br>2. Optional to add rock sugar	Formula passed on from a Chinese Medicine Physician 40 years ago
	Chrysanthemum morifolium Ramat.	T5897	12.5		8.38		
	Prunella vulgaris L.	T5898	23.75		15.93		
	Lysimachia Christinae Hance	T5899	14.12		9.47		
	Dioscorea hypoglauca Palibin	T5900	11		7.38		
	Microcos paniculata L.	T5901	33.71		22.61		
KL10	*Bombax ceiba* L.	T5902	50.3	130.14	38.65	Boil with 5-6 bowls of water for 50 minutes until 2-3 bowls of water left	The stall owner considers *A. capillaris* as floral herb
	Plumeria rubra L.	T5903	11.95		9.18		
	Chrysanthemum morifolium Ramat.	T5904	10.74		8.25		
	Pueraria lobata (Willd.) Ohwi	T5905	8.88		6.82		
	Artemisia capillaris Thunb.	T5906	31.59		24.27		
NT01	*Bombax ceiba* L.	T5907	31.14	112.64	27.65	1. Bring to a boil with 8 bowls of water and brew over low heat for 30 minutes<br><br>2. Optional to add brown slab sugar or *S. grosvenorii*	Moist herbs, insects found
	Plumeria rubra L.	T5908	5.31		4.71		
	Chrysanthemum morifolium Ramat.	T5909	6.8		6.04		
	Pueraria lobata (Willd.) Ohwi	T5910	3.84		3.41		
	Artemisia capillaris Thunb.	T5911	25.42		22.57		
	Prunella vulgaris L.	T5912	15.21		13.5		
	Glycyrrhiza uralensis Fisch.	T5913	10.1		8.97		
NT02	*Bombax ceiba* L.	T5914	54.28	152.38	35.62	1. Bring up a boil with 6-10 bowls of water and brew over	1. Should be taken as warm decoction
	Plumeria rubra L.	T5915	3.19		2.09		
	Chrysanthemum morifolium Ramat.	T5916	9.55		6.27		

	Lonicera japonica Thunb.	T5917	2.22		1.46	low heat for 45-60 minutes<br><br>2. Can add sugar to the tea	2. Persons who are of cold constitution can only drink half bowl<br><br>3. Should be stored in refrigerator after boiling<br><br>4. Moist herbs, insects found
	Artemisia capillaris Thunb.	T5918	35.81		23.5		
	Prunella vulgaris L.	T5919	20.04		13.15		
NT03	*Bombax ceiba* L.	T5920	33.99	157.05	21.64	1. Brew over medium heat with 10 bowls of water for 45 minutes<br><br>2. Optional to add rock sugar	The stall owner claimed that the tea is suitable for everyone but subject to the amount of consumption
	Plumeria rubra L.	T5921	10.13		6.45		
	Chrysanthemum morifolium Ramat.	T5922	9.31		5.93		
	Lonicera japonica Thunb.	T5923	6.16		3.92		
	Pueraria lobata (Willd.) Ohwi	T5924	5.87		3.74		
	Artemisia capillaris Thunb.	T5925	31.8		20.25		
	Prunella vulgaris L.	T5926	27.66		17.61		
NT04	*Bombax ceiba* L.	T5927	34.17	106.07	32.21	1. Brew with 10 bowls of water for 30-60 minutes<br><br>2. Add sugar	Moist herbs, insects found
	Plumeria rubra L.	T5928	35.93		33.87		
	Chrysanthemum morifolium Ramat.	T5929	23.56		22.21		
	Lonicera japonica Thunb.	T5930	25.37		23.92		
	Sophora japonica L.	T5931	24.42		23.02		
NT05	*Bombax ceiba* L.	T5932	37.36	185.39	20.15		

	Chrysanthemum morifolium Ramat.	T5933	5.6		3.02	Bring to a boil with 8-10 bowls of water and brew over low heat for 60 minutes	1. Persons of cold constitution should beware of overdose<br><br>2. Moist herbs, insects found
	Lonicera confusa DC.	T5934	1.85		1		
	Artemisia capillaris Thunb.	T5935	18.59		10.03		
	Prunella vulgaris L.	T5936	10.44		5.63		
	Polygonum chinense L.	T5937	16.49		8.89		
	Desmodium styracifolium (Osh.) Merr.	T5938	24.68		13.31		
NT06	*Bombax ceiba* L.	T5939	12.73	142.14	8.96	Bring to a boil with 3 litre of water and brew for 60 minutes	1. Their formula is good for the skin<br><br>2. Not that cold when compared to Xiasangju (another heat-clearing herbal tea)<br><br>3. Moist herbs, insects and mould found
	Chrysanthemum morifolium Ramat.	T5940	2.27		1.6		
	Artemisia capillaris Thunb.	T5941	8.46		5.95		
	Prunella vulgaris L.	T5942	16.65		11.71		
	Morus alba L.	T5943	79.43		55.88		
	Tetrapanax papyrifer (Hook.) K.Koch	T5944	0.75		0.53		
	Poria cocos (Schw.) Wolf	T5945	9.87		6.94		
NT07	*Bombax ceiba* L.	T5946	34.54	154.48	22.36	1. Brew over medium heat with 10-12 bowls of water for 60 minutes	Suitable for everyone as it is not very cold in nature
	Chrysanthemum morifolium Ramat.	T5947	11.02		7.13		
	Artemisia capillaris Thunb.	T5948	54.95		35.57		
	Prunella vulgaris L.	T5949	32		20.71		

	Tetrapanax papyrifer (Hook.) K.Koch	T5950	1.32		0.85	2. Rinse the herbs before boiling	
NT08	*Bombax ceiba* L.	T5951	38.05	128.56	29.6	Brew with 6-7 bowls of water for 30-90 minutes	Suitable for everyone to drink
	Plumeria rubra L.	T5952	6.03		4.69		
	Chrysanthemum morifolium Ramat.	T5953	8.95		6.96		
	Lonicera japonica Thunb.	T5954	6		4.67		
	Pueraria lobata (Willd.) Ohwi	T5955	5.46		4.25		
	Artemisia capillaris Thunb.	T5956	22.66		17.63		
	Prunella vulgaris L.	T5957	13.45		10.46		
NT09	*Bombax ceiba* L.	T5958	42.4	151.77	27.94	Brew with 6-7 bowls clear water for 40 minutes. Until 2-3 bowls of tea left	People with G6PD deficiency should avoid drinking this tea.
	Plumeria rubra L.	T5959	3.25		2.14		
	Chrysanthemum morifolium Ramat.	T5960	14.14		9.32		
	Lonicera japonica Thunb.	T5961	4.3		2.83		
	Artemisia capillaris Thunb.	T5962	44.94		29.61		
	Prunella vulgaris L.	T5963	26.13		17.22		
NT10	*Bombax ceiba* L.	T5964	18.86	96.4	19.56	1. Bring to a boil with 10 bowls of water, turn to medium heat for 1 hour until 4 bowls left.<br><br>2. Optional to add S. grosvenorii, sugar,	1. Moist herbs, insects found<br><br>2. Suitable for everyone
	Chrysanthemum morifolium Ramat.	T5965	11		11.41		
	Lonicera japonica Thunb.	T5966	4.15		4.3		
	Pueraria lobata (Willd.) Ohwi	T5967	8.79		9.12		
	Prunella vulgaris L.	T5968	5.31		5.51		

						brown slab sugar or candied jujubes	
NT11	*Bombax ceiba* L.	T5969	55.96	179.47	31.18	Bring to a boil with 3 litre of water, and then turn to low heat for 45 minutes	Not suitable for pregnant women
	Plumeria rubra L.	T5970	18.16		10.12		
	Chrysanthemum morifolium Ramat.	T5971	18.56		10.34		
	Lonicera japonica Thunb.	T5972	6.99		3.89		
	Artemisia capillaris Thunb.	T5973	36.23		20.19		
	Desmodium styracifolium (Osh.) Merr.	T5974	16.75		9.33		
