# Supplementary material for: Ethnopharmacology of five flowers herbal tea, a popular traditional beverage in Hong Kong and South China
Source: J Ethnobiol Ethnomed. 2024 Mar 15;20:36. doi: 10.1186/s13002-024-00674-z (PMC10943788; doi:10.1186/s13002-024-00674-z)
Supplement: Supplementary file 2 — Additional file 2: Herbal tea ingredients identified in Five Flowers Tea samples and their pharmacological properties. [file 13002_2024_674_MOESM2_ESM.pdf]

Additional file 2: Herbal tea ingredients identified in Five Flowers Tea samples and their pharmacological properties

Family	Botanical name [14, 54] [updated name][55]	Local name	Plant part used	Prevalence in sample	Medicinal use	Ethnomedical action and indication [14]	Reference
**Asteraceae**	*Artemisia capillaris* Thunb.	綿茵陳	aerial portion	28	Anti-inflammation, anti-cancer, hepatitis, jaundice, hepatoprotective	To clear and drain dampness-heat, disinhibit gallbladder and abate jaundice. Apply to jaundice, scanty urination, dampness-warmth, summerheat-dampness, and itching caused by dampness sore.	[17-19]
**Malvaceae**	*Gossampinus malabarica* (DC.) Men.<br><br>[*Bombax ceiba* L.]	木棉花	flower	33	Anti-inflammation, anti-diarrheal, diuretic, cancer, hepatoprotective, cardioprotective	To clear heat, drain dampness, and remove toxin. Apply to diarrhea, dysentery, and hemorrhoids bleeding.	[56, 57]
**Asteraceae**	*Chrysanthemum morifolium* Ramat.	菊花	flower	32	Anti-inflammation, anti-microbial, anti-viral, cancer, hepatoprotective, neuroprotective	To disperse wind, clear heat, pacify the liver, improve vision, clear heat and remove toxin. Apply to common cold caused by wind-heat, headache and dizziness, red painful swelling eyes, blurry vision, sore, abscess, and skin infections.	[58, 59]

**Dioscoreaceae**	*Dioscorea hypoglauca* Palibin	萆薢	rhizome	1	Anti-microbial, anti-inflammation, immunomodulation	To drain dampness, dispel turbidity, dispel wind and relieve impediment. Apply to unctuous strangury with white turbid unie, profuse white vaginal discharge, painful *bi* disorder caused by wind-dampness, inhibited joint, aching lower back and knees	[60]
**Rosaceae**	*Eriobotrya japonica* (Thumb.) Lindl.	枇杷葉	leaf	1	Anti-inflammation, diabetes, cancer, bacterial infection, diuretic, hepatoprotective, anti-viral, liver function improvement	To clear the heat, suppress cough, downbear counterflow, and stop vomiting. Apply to cough caused by lung-heat, suppress cough, downbear counterflow, vomiting and hiccup caused by stomach heat, heat vexation stop thirst.	[61, 62]
**Fabaceae**	*Glycyrrhiza uralensis* Fisch.<br><br>[*Glycyrrhiza uralensis* Fisch. ex DC.]	甘草	roots and rhizomes	5	Anti-inflammation, anti-tumor, hepatoprotection, anti-bacterial, anti-viral, heartburn, gastritis	To tonify spleen and qi, clear heat, remove toxin, dispel phlegm, suppress cough, relax spasm, relieve pain, and moderate drug actions. Apply to spleen-stomach weakness, fatigue, lack of strength, palpitations, shortness of breath, cough and profuse sputum, painful spasm in the stomach duct, abdomen and limbs, swelling abscess, sore, skin infections, and reducing the other drugs' toxin and drastic action.	[63, 64]

**Poaceae**	*Imperata cylindrica* Beauv.var.major (Nees) C.E.Hubb.<br><br>[*Imperata cylindrica* (L.) Raeusch.]	白茅根	rhizome	1	Anti-inflammation, anti-hypertensive, anti-bacterial, Anti-cancer, immunomodulation	To cool the blood, stanch bleeding, clear heat, and promote urination. Apply to hematemesis, epistaxis and hematuria caused by blood heat, vexation and thirst caused by heat disease, dampness-heat jaundice, edema, small quantity of urination, and heat strangury with slow pain.	[65, 66]
**Caprifoliaceae**	*Lonicera japonica* Thunb.	金銀花	flower	23	Anti-inflammation, anti-viral, anti-tumor, anti-bacterial, hepatoprotective	To clear heat, remove toxin, and disperse wind-heat. Apply to swelling abscess, deep-rooted boil, sore, throat *bi* disorder, erysipelas, heat-toxin blood dysentery, common cold caused by wind-heat, and fever in warm disease.	[67]
**Primulaceae**	*Lysimachia christinae* Hance	金錢草	whole herb	4	Anti-ancer, neuroprotective, hepatoprotective, anti-cholecystitis, immunity strengthening	To drain dampness, abate jaundice, disinhibit urine, relieve stranguria, remove toxin, and relieve swelling. Apply to dampness-heat jaundice, gallbladder distention, hypochondriac pain, stone strangury, heat strangury, difficult and painful urination, swelling abscess, deep-rooted boil, sore, and bites of insect, worm or snack.	[68-71]

**Malvaceae**	*Microcos paniculata* L.	布渣葉	leaf	2	Anti-diarrheal, anti-microbial, anti-inflammation, cardioprotective, hepatoprotective, jaundice	To promote digestion, resolve stagnation, clear heat, and drain dampness. Apply to food retention, common cold with fever, and dampness-heat jaundice.	[72]
**Moraceae**	*Morus alba* L.	桑葉	leaf	3	Anti-microbial, anti-diabetic, anti-diarrheal, anti-cancer, cardioprotective, anti-atherosclerotic, immunomodulatory, nephroprotective, hepatoprotective	To disperse wind-heat, clear the heat, moisten dryness, clear the liver, and improve vision. Apply to common cold caused by wind-heat, lung heat, dryness cough, dizziness, headache, red eyes, and blurry vision.	[73,74]
**Bignoniaceae**	*Oroxylum indicum* (L.) Vent.<br><br>[*Oroxylum indicum* (L.) Kurz]	木蝴蝶	seed	3	Anti-inflammation, anti-cancer, anti-hyperglycemia, antibacterial, neurogenesis, wound healing	To clear the heat, soothe the throat, pacify the liver, and harmonize the stomach. Apply to cough caused by lung-heat, throat *bi* disorder, hoarse voice, pain in liver and stomach due to qi stagnation.	[75]
**Apocynaceae**	*Plumeria rubra* L.	雞蛋花	flower	22	Anti-inflammation, anti-diabetic, anti-microbial, anti-pyretic, anti-cancer,	To clear heat and drain dampness, detoxify and soothe the lung. Apply to damp-heat diarrhea, tenesmus and lung heat cough.	[76,77]

					anti-viral, gastroprotective		
**Polygonaceae**	*Polygonum chinense* L.<br><br>[*Persicaria chinensis* (L.) H.Gross]	火炭母	whole herb	4	Anti-inflammation, anti-microbial, anti-bacterial, anti-cancer, gastroprotective	To clear heat and drain dampness, detoxify and cool the blood. Apply to damp-heat diarrhea, dysentery, jaundice, sore throat and damp-heat skin rash.	[78-80]
**Polyporaceae**	*Poria cocos* (Schw.) Wolf<br><br>[*Wolfiporia extensa* (Peck) Ginns]	茯苓	sclerotium	2	Anti-inflammation, anti-cancer, immunomodulatory, hepatitis, antidiabetic, anti-hypertensive, anti-nephritic	To promote urination to drain dampness, fortify the spleen, and calm the heart. Apply to edema, small quantity of urination, dizziness and palpitations caused by phlegm-fluid retention, spleen deficiency, reduced food intake, sloppy stool, diarrhea, disquieted heart spirit, fright palpitations and insomnia.	[81]
**Lamiaceae**	*Prunella vulgaris* L.	夏枯草	fruit spike	21	Anti-inflammation, anti-pyretic, anti-rheumatic, anti-viral, immunomodulatory, anti-hypertensive, hypoglycemic, hepatoprotective	To clear the liver, purge fire, improve vision, dissipate bind and disperse swelling, Apply to red painful swelling eyes, eyeball pain at night, headache dizziness, scrofula, goiter, acute mastitis, mammary	[82]

						hyperplasia, and distending pain in the breasts.	
**Fabaceae**	*Pueraria lobata* (Willd.) Ohwi<br><br>[*Pueraria montana* var. *lobata* (Willd.) Maesen & S.M.Almeida ex Sanjappa & Predeep]	葛花	flowers	8	Anti-inflammation, anti-cancer, anti-pyretic, analgesic, hepatoprotective, antidiabetic, neuroprotective, cardioprotective, nephroprotective, immunomodulatory	To enliven the spleen and sober up, release the flesh and clear the heat, engender the fluid and quenching the thirst, stop diarrhea and treat dysentery. Apply to fever with thirst and agitation, loss of appetite, nausea and vomiting with acid regurgitation, hematemesis, bloody stool.	[83]
**Smilacaceae**	*Smilax glabra* Roxb.	土茯苓	rhizome	1	Anti-inflammation, anti-infective, anti-viral, anti-cancer, detoxification, cardioprotection, hepatoprotective, nephroprotective, immunomodulatory	To remove toxin, remove dampness, relieve and facilitate joints. Apply to spasm of joints, pain in the sinews and bones caused by syphilis or mercury poisoning; dampness-heat strangury, turbid, vaginal discharge, swelling abscess, scrofula, scabies, and tinea.	[84, 85]
**Fabaceae**	*Sophora japonica* L.	槐花	flower	5	Anti-inflammation, anti-osteoporotic, anti-tumor, anti-bacterial, anti-viral, anti-atherosclerotic	To cool the blood, stop bleeding, clear the liver and purge fire. Apply to bloody stool, hemorrhoid bleeding, bleeding, blood dysentery, menstrual flooding and spotting, hematemesis,	[86]

	[*Styphnolobium japonicum* (L.) Schott]					epistaxis, red eyes caused by liver-heat, headache and dizziness.	
**Araliaceae**	*Tetrapanax papyrifer* (Hook.)K.Koch	通草	stem pith	2	Anti-inflammation, anti-thrombin, anti-hepatotoxic	To clear heat and promote urination, regulate qi to promote lactation. Apply to damp-heat strangury, edema with oliguria and retained breastmilk.	[87]
